# Incidence and severity of postoperative sore throat: a randomized comparison of Glidescope with Macintosh laryngoscope

**DOI:** 10.1186/s12871-017-0421-4

**Published:** 2017-09-12

**Authors:** Mansoor Aqil, Mueen Ullah Khan, Saara Mansoor, Saad Mansoor, Rashid Saeed Khokhar, Abdul Sattar Narejo

**Affiliations:** 1 Department of Anesthesiology, King Saud University Medical City, P.O Box 7805, Riyadh, 11472 Saudi Arabia; 2 College of Medicine, Alfaisal University, Riyadh, Saudi Arabia; 3 Department of Anesthesiology, King Khalid Hospital, Hail, Saudi Arabia

**Keywords:** Sore throat, Laryngoscope, Peroperative complications, Endotracheal intubation

## Abstract

**Background:**

Postoperative sore throat (POST) is a common problem following endotracheal (ET) intubation during general anesthesia. The objective was to compare the incidence and severity of POST during routine intubation with Glidescope (GL) and Macintosh laryngoscope (MCL).

**Methods:**

One hundred forty adult patients ASA I and II with normal airway, scheduled to undergo elective surgery under GA requiring ET intubation were enrolled in this prospective randomized study and were randomly divided in two groups, GL and MCL. Incidence and severity of POST was evaluated at 0, 6, 12 and 24 h after surgery.

**Results:**

At 0 h, the incidence of POST was more in MCL than GL (*n* = 41 v.s *n* = 22, *P* = 0.001), and also at 6 h after surgery (*n* = 37 v.s *n* = 23, *P* = 0.017). Severity of POST was more at 0, 6 and 12 h after surgery in MCL (*P* < 0.001, *P* = 0.001, *P* = 0.004 respectively).

**Conclusions:**

Routine use of GL for ET tube placement results in reduction in the incidence and severity of POST compared to MCL.

**Trial regisration:**

ClinicalTrials.gov NCT02848365. Retrospectively Registered (Date of registration: July, 2016).

## Background

Postoperative sore throat (POST) is a common problem following the use of endotracheal (ET) intubation during general anesthesia (GA) [1]. It leads to dissatisfaction and discomfort after surgery and can delay a patient’s return to normal routine activities [2]. POST has been rated by patients as the eighth most adverse effect in the postoperative period [3]. The incidence of POST after ET intubation varies from 14.4–90% [1, 4, 5].

The method of airway management is an important factor in causation of POST [1]. ET intubation is commonly performed under direct laryngoscopy using Macintosh laryngoscope (MCL). To visualize glottis with this laryngoscope, a forward and upward force is applied on the handle of the laryngoscope to align visual, oral, pharyngeal and laryngeal axes. The force is also transferred to the arytenoid cartilages of the larynx and may damage the mucosa of delicate airway that may cause trauma to the glottis resulting in POST [1]. Glidescope (GL) is a video laryngoscope (VL) and requires minimal head manipulation and positioning and allows rapid visualization of the larynx, compared to conventional direct laryngoscopy with MCL [6] and provides improved view of the glottis that facilitates ET intubation [7]. It has been found to be less traumatic than MCL due to avoidance of the forces applied on the laryngoscope blade [8, 9].

As ET intubation using GL requires less force during visualization of glottis [8] and has been found to be faster with superior view [7], we hypothesized that ET intubation with GL would cause less trauma to the throat and consequently, lower incidence of POST. However, on search of literature we found contrasting results regarding the incidence of POST while using GL and MCL [10–13]. We designed this prospective randomized study to find out and compare the incidence and severity of POST while using MCL and GL in our institution in patients with normal airway during their routine usage.

## Methods

**Fig. 1:**
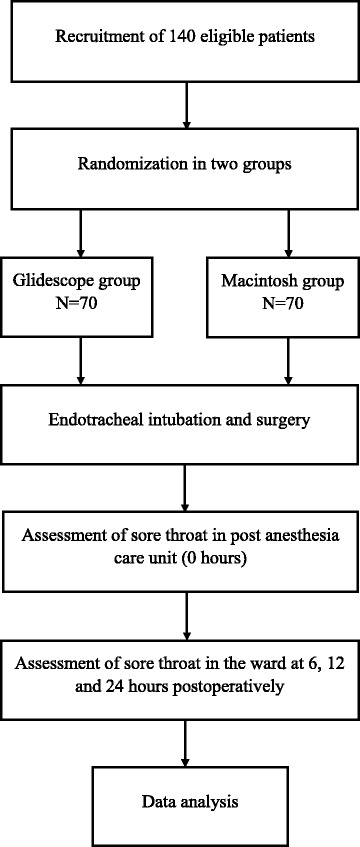
Title: Study Consort

After approval of the Institutional Review Board of King Khalid University hospital, Riyadh, Saudi Arabia, this prospective randomized study was conducted during the period January 2012–January 2017. Study consort is shown in Fig. 1.

Clinical trial registration: ClinicalTrials.gov, NCT02848365.

### Inclusion criteria

After signing of the informed consent, 140 adult patients between age 18–60 years, ASA physical status I and II and Mallampati class 1& 2, BMI < 35 kg/m^2^, undergoing elective surgical procedures (not exceeding two hours in duration) requiring ET intubation were included in the present study.

### Exclusion criteria

Patients undergoing day case, bariatric, cardiac, nasal, oral or head & neck surgeries, requiring placement of throat pack or nasogastric/ orogastric tube, patients assigned to rapid sequence induction, hoarseness, patients with anticipated difficult intubation, history of recent upper respiratory tract infection, history of difficult intubation and psychiatric disorders hindering proper evaluation, use of steroids (oral or inhalational) or non-steroidal anti-inflammatory drugs within one week of surgery or previous surgery within last two weeks were excluded.

### Method of randomization

The randomization scheme was generated through online software using the (www.randomizer.org).

One day before surgery, the patients were explained how to rate the severity of POST by using a 10 point score: 0 = no sore throat, 1–3 = mild sore throat (complains of sore throat only on asking), 4–7 = moderate sore throat (complains of sore throat on his/her own), and 8–10 = severe sore throat (change of voice or hoarseness, associated with throat pain) [14]. The incidence of POST was recorded using a yes/no questionnaire.

All the patients were fasting from midnight and received premedication orally with 2 mgs lorazepam, 150 mg ranitidine and 10 mg metoclopramide two hours before surgery with a sip of water. Group MCL was intubated with Macintosh laryngoscope, Group GL was intubated with Glidescope. All the ET intubations were done by anesthesiologists who were having the experience of at least 100 intubations with each instrument.

In the operating room, a peripheral intravenous (i.v.) line was established and standard monitors like continuous ECG, oxygen saturation by pulse oximetry (SPO_2_) and non-invasive blood pressure (NIBP) were applied. Patient’s head was placed on a 4–6 inches. high pillow to make a “sniffing morning air position”. Before induction of anesthesia, the patients were preoxygenated with 100% oxygen for 3 min and received i.v. dexamethasone in the dose of 8 mg. GA was induced with i.v. propofol in the dose 2 mg/kg and fentanyl 2 microgram (mcg)/kg. For ET intubation, neuromuscular block was attained with cisatracurium in the dose of 0.15 mg/kg. Till the establishment of adequate neuromuscular block, the patients’ ventilation was assisted manually using a face mask. During this period the patients’ lungs were ventilated with 100% oxygen containing 2% sevoflurane on a circle breathing system. After establishment of adequate neuromuscular block confirmed by absence of response to train of four stimulation (TOF) of ulnar nerve, the trachea was intubated using either MCL or GL (according to the patients’ group). Cuffed poly vinyl chloride ET tube was used in all patients and the size was 7.0 mm internal diameter (I.D) in female patients and size 8.0 mm I.D in male patients. The ET tube was lubricated with water soluble KY® Jelly only. After placement, the cuff of the ET tube was inflated gradually with air until an intra-cuff pressure of 20–25 cm H_2_O (measured with aneroid manometer VBM, Sulz, Germany) and there was no audible leakage with peak airway pressure of 20 cm H_2_O. Heat and moisture exchangers were used in the gas delivery circuit in all cases. For intubation with GL, we used size 4 scope and for patients in MCL group, size 4 blade was used. The allowed time for successful ET intubation was up to two minutes. If the time to intubation (TTI) exceeded 2 min or SPO_2_ dropped below 92%, the patient’s lungs were ventilated with 100% oxygen containing 2% sevoflurane for 30 seconds (s) and a second intubation trial was attempted. Manual intermittent positive pressure ventilation was done between the attempts with the same anesthetic mixture. A maximum of two intubation attempts were allowed. If two attempts were unsuccessful in proper ET placement, failed intubation algorithm was followed [15].

After successful ET placement, GA was maintained with 2–3% end tidal concentration of sevoflurane in 50% oxygen air mixture. Nitrous oxide was not used in the study. Intraoperative analgesia was maintained with intermittent boluses of fentanyl in the dose of 1 mcg/kg when heart rate or NIBP increased by 20% or more from the baseline reading. Cisatracurium was repeated in the dose of 2 mg bolus on appearance of 2 twitches on TOF stimulation of ulnar nerve. All of the patients received i.v. paracetamol in the dose of 1 g intraoperatively. The remaining course of GA was on the anesthesiologist’s discretion. On completion of the surgery, the residual neuromuscular block was reversed with the mixture of 2.5 mg neostigmine and glycopyrolate 0.4 mg administered i.v. On attaining adequate breathing, the oral cavity was cleared of any secretions with the help of a flexible soft suction catheter size 12F and the trachea was extubated at T4/T1 ratio of >90%.

In the operating room, the anesthesiologist recorded the age, gender, height, weight, smoking habits, patient’s group, type & duration of surgery, TTI, number of attempts required for intubation, need for external pressure during intubation, view of the glottis based on Cormack and Lehane’s (C&L) classification and percentage of glottic opening (POGO) score [16]. The presence of blood stain on intubating instrument was also recorded.

On admission to post anesthesia care unit (PACU), a blinded observer asked the patients to rate their POST and was labelled as time 0 h and subsequently in the ward at 6 h and 12 h after surgery and also on first post-operative day at about 24 h after surgery. For post-operative pain relief all of the patients received morphine patient controlled analgesia (PCA) with 2 mg bolus and 10 min lockout time and total morphine consumption in 24 h was recorded. Additionally, all of the patients received i.v. paracetamol (1 g 6 hourly) and injection lornoxicam (8 mg slow i.v. 12 hourly) on regular basis for first 24 h.

#### Outcome measures

The primary outcome measure of the study was the incidence of POST during first 24 h in adult patients undergoing surgery under GA. The secondary outcome measure was the severity of POST in 24 h after surgery. Additional outcome measure were view of the glottis based on POGO score and C&L classification, TTI, number of attempts required for intubation, patient’s satisfaction score [2] and intubation difficulty score (IDS) [10]. An IDS >5 was considered a difficult intubation [17].

#### Sample size

Sample size calculation was based on incidence of POST which was our primary outcome measure. With a power of 80% and alpha level of 0.05 for 2-tailed statistical analysis and estimated incidence POST of 0.46, and 0.17 [18] in the two groups, a sample size of 69 patients for each group was calculated as being appropriate. Considering the possible dropouts, 70 patients were enrolled in each group.

### Statistical analysis

The data was analyzed with SPSS version 22 Statistics™ (IBM SPSS Inc., Chicago, IL, USA). The outcome measures are presented in tables as either mean ± SD or as percentages. Student’s t-Test was used to analyze the difference between the two groups for demographics (age, weight, height and BMI), baseline characteristics (mouth opening, thyromental distance) and other variables (TTI, duration of surgery, POGO score, total dose of fentanyl, lowest saturation and total dose of morphine PCA concentration). Pearson’s Chi Square test was used to analyze the difference between the two groups for gender, need of external pressure, number of attempts needed for successful intubation, cigarette smoking, incidence of POST and presence of blood stain on instrument. Wilcoxon Mann- Whitney U test was used to analyze the difference between the two groups for severity of sore throat, Mallampati class, overall IDS, C&L score and patient satisfaction score. A *p* value <0.05 was considered as statistically significant.

## Results

Of 140 patients of both the groups, all completed the study and no patient was excluded. The demographic data is shown in Table 1. The groups were similar in the characteristics like age, gender, weight, height, BMI, preoperative anesthesia assessment data and smoking habits of the patients (Table 1). There was no difference in duration of surgery, total intraoperative consumption of fentanyl, lowest SPO_2_ during intubation and total dose of morphine PCA required during first 24 h after surgery (Table 2).

**Table 1:** Demographic and preoperative airway assessment data

	Glidescope (*N* = 70)	Macintosh (*N* = 70)	*P* value
Age (years)	36.8 ± 10.9	38.6 ± 10.7	0.33
Weight (kg)	74.9 ± 10.5	73.0 ± 11.7	0.31
Height (cm)	164.2 ± 7.5	165.1 ± 8.0	0.51
BMI (kg/m^2^)	27.8 ± 3.7	26.9 ± 4.1	0.14
Gender (Male/ Female)	33/37	26/44	0.23
Cigarette smoking (Yes/ No)	15/55	20/50	0.33
Mouth opening (mm)	49.3 ± 9.8	50.0 ± 9.6	0.64
Thyromental distance (cm)	8.8 ± 1.3	8.5 ± 1.1	0.13
Mallampati Class (1/2)	31/39	35/35	0.5

Values expressed as mean ± SD where applicable

**Table 2:** Comparison of laryngoscopic scores, intubating data, duration of surgery and intraoperative and postoperative analgesia

	Glidescope (*N* = 70)	Macintosh (*N* = 70)	*P* value
Duration of surgery (min)	82.6 ± 25.3	86.5 ± 30.5	0.42^a^
Time to intubate (s)	36.1 ± 12.9	47.8 ± 28.1	0.002^a^
POGO score (%)	77.1 ± 28.9	43.4 ± 28.2	<0.001^a^
C& L score 1/2/3/4	35/32/3/0	18/38/14/0	<0.001^b^
Total dose of intraoperative fentanyl consumption (micrograms)	190.1 ± 36.8	200.8 ± 42.5	0.12^a^
Lowest saturation during intubation (%)	94.8 ± 2.0	94.3 ± 1.6	0.08^a^
Total dose of Morphine PCA consumed in first 24 h	18.0 ± 7.2	18.9 ± 9.9	0.53^a^
Need of external pressure (Yes/No)	21/49	37/33	0.006^c^
Number of attempts needed for intubation (1/2)	64/6	55/15	0.005^c^
Blood stain on the instrument (Yes/No)	13/57	21/49	0.115^c^
Number of patients with sore throat (Yes/No)			
At 0 h	22/48	41/48	0.001 ^c^
6 h	23/47	37/33	0.017 ^c^
12 h	20/50	31/39	0.053 ^c^
24 h	7/63	15/55	0.063 ^c^
Intubation difficulty score. Median (interquartile range)	2.73 (3)	4.16 (4)	<0.001^b^
Patient Satisfaction score. Median (interquartile range)	8.0 (2)	5.0 (2)	<0.001^b^

Values expressed as mean ± SD or median (interquartile range) where applicable

^a^Student’s t-Test

^b^Wilcoxon Mann-Whitney U Test

^c^Pearson’s Chi Square Test

**Fig. 2:**
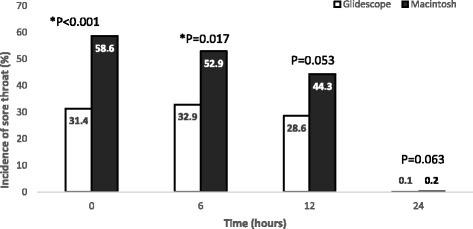
Title: Incidence of sore throat at different time intervals. Legend: * *P* < 0.05 during intergroup comparison

Figure 2 shows the comparison of incidence of POST in both groups at 0,6,12 and 24 h after surgery. At 0 h, the incidence of POST was higher in MCL than GL (*n* = 41 v.s *n* = 22, *P* = 0.001) and also at 6 h after surgery (*n* = 37 v.s *n* = 23, *P* = 0.017); however, there was no significant difference in the incidence of POST at 12 h (*n* = 31 v.s *n* = 20, *P* = 0.053) and at 24 h (*n* = 15 v.s *n* = 7, *P* = 0.063).

**Fig. 3:**
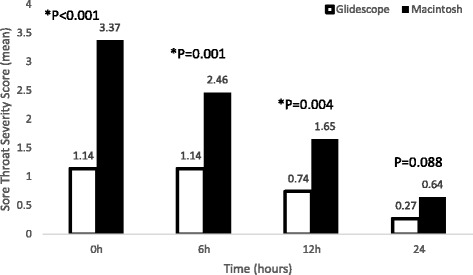
Title: Severity of sore throat at different time intervals. Legend: * P < 0.05 during intergroup comparison

As shown in Fig. 3, the severity of POST was more at 0, 6 and 12 h after surgery in MCL group compared to GL group (*P* < 0.001, P = 0.001, *P* = 0.004 respectively). However, there was no significant difference in the severity of POST at 24 h between the two groups (*P* = 0.088).

As shown in Table 2, TTI was less in the GL group compared to MCL group (36.1 ± 12.9 v.s 47.8 ± 28.1, *P* = 0.002). During ET intubation, GL provided superior view of the glottis compared to MCL as assessed by POGO score (77.1 ± 28.9 v.s 43.4 ± 28.2, *P* < 0.001) and C&L view of the glottis (P < 0.001). The need of external pressure on the neck for visualization of the glottis was less with GL compared to MCL (*n* = 21 v.s *n* = 37, *P* = 0.006). Using GL, successful ET placement was achieved in first attempt in majority of the patients compared to MCL (*n* = 51 v.s *n* = 38, *P* = 0.022). There was no significant difference in the duration of surgery, total intraoperative consumption of fentanyl, total morphine PCA consumption in first 24 h, lowest SPO_2_ during intubation and the presence of blood stain on the instrument.

Table 2 shows the comparison of IDS using both instruments for intubation. The IDS was significantly higher in MCL group compared to GL group (median = 6.0 v.s 3.0 respectively, P < 0.001). Patients’ satisfaction scores were significantly higher in the GL group (median = 8.0) as compared to the MCL group (median = 8.0 v.s 5.0 respectively, P < 0.001).

## Discussion

Numerous factors contribute in the causation of POST e.g. gender, age, type of muscle relaxant used, gynaecological surgery, ET tube size, cuff design, high cuff pressure, smoking habit and duration of surgery [4, 19–22]. In our study we did not find any contribution from these factors as both the groups were similar in this respect.

Our main results show that (a) overall incidence of POST was high with MCL compared to GL, (b) incidence of POST was more at 0 h and at 6 h with MCL compared to GL, however, there was no difference between the groups in the incidence at 12 and 24 h after surgery, (c) severity of POST was more with MCL at 0, 6 and 12 h with MCL, (d) C&L grade of view of the glottis was higher and IDS was more with MCL compared to GL, (e) need of external pressure on the neck for successful intubation was more with MCL, (f) POGO score was higher with GL, (g) TTI required for successful intubation was more with MCL and (h) patient’s satisfaction score was comparatively more with GL.

Similar to our results, Najafi et al. [4] compared incidence of POST after laryngoscopy with GL and MCL in 300 patients and found it to be higher in MCL group. Despite similarities in results, there are certain divergences in the design of both the researches; e.g. the authors studied incidence of POST for 48 h and included the patients undergoing comparatively prolonged surgeries (average surgery duration was 130 min), while we studied POST for only 24 and the average duration of surgery was 85 min. In contrast to our results, Najafi et al. [4] found lower incidence of POST at 0, 6, 12, 24 h with both the devices. They did not specify the experience of the intubating person or the BMI of the patients, neither did they compare the number of attempts needed for successful intubation. The probable reason of lower incidence of POST in their study may be due to relatively more experienced anesthesiologists performing ET intubation or the patients having lower BMI or that fewer number of attempts were required for successful intubation. Moreover, our research adds to Najafi’s results by studying important additional factors like smoking habits, POGO score, C&L score, presence of blood stain on the intubating instrument, intubation difficulty score (all of which may affect the incidence and severity of POST) and the ultimate goal, “patient’s satisfaction score”.

One of the major causes of POST is trauma to the airway mucosa during airway management. The degree of mucosal damage is related to amount of forces applied during laryngoscopy and the number of attempts required for successful intubation [1, 23]. Using GL for laryngoscopy and ET intubation, alignment of laryngeal and oral axis is not required due to unique design of its blade which has a camera at its distal end. Russell et al. compared the forces applied during the laryngoscopy while using GL and MCL. Their results showed lower peak force with GL in comparison to MCL (9 N v.s 20 N; *P* = 0.0001) [8]. As less amount of force is required during laryngoscopy using GL, it produced less trauma to the mucosa of the airway, resulting in lower incidence and severity of POST in our study. In a Cochrane database systemic review, Lewis et al. [24] compared VL and direct laryngoscopy for endotracheal intubation and concluded that VL reduces laryngeal and airway trauma. Although, in our study, the number of patients with blood stain on the intubating device was not statistically high in MCL group, nevertheless, they were higher in number compared to GL group patients. The reason for intubating trauma not showing on the intubating instrument may be because blood on the device would only appear if it caused trauma directly, however, the trauma caused by the ET tube would not show on the intubating instrument. Secondary to inappropriate glottic visualization (higher C&L grades and lower POGO score) in the MCL group, the ET tube probably scrubbed against the glottic or laryngeal mucosa and traumatized it resulting in higher incidence of POST.

Another factor contributing to POST is the duration of laryngoscopy and TTI [11]. Our results show that TTI was higher with MCL compared to GL which contributed to higher incidence of POST in this group. The reason of greater TTI with MCL was comparatively poor view of the glottis based on C&L classification and POGO scores which required external manipulation of the neck during ET intubation. Similar to our results Hsu et al. found higher incidence of POST with MCL in comparison to GL following double lumen tube insertion. They related the higher incidence of POST to longer TTI required using MCL compared to GL (62.5 s ± 29 s v.s 45.6 s ± 10.7 s (*P* = 0.007) [11]. Similarly Johns P et al. also found higher incidence of POST with MCL compared to GL during routine nasotracheal intubation. Their TTI for successful ET intubation was also more with MCL compared to GL (43.5 s v.s. 23.2 s, *P* = 0.0023) [10]. Contrary to our results, Cirilla D and colleagues did not find any difference in the incidence of POST during routine use of GL and MCL in patients with normal airway (32.4% v.s. 36.3%, *P* = 0.619) [12]. In their study, ET intubation was performed by residents, nurses and anesthesiologists. Furthermore, the authors did not record and compare the TTI in their study, in our opinion, it is difficult to compare our results with their study since we found significantly more TTI with MCL compared to GL during visualization of the glottis and placement of ET tube into the trachea which resulted in more trauma to mucosa of the airway and the incidence and severity of POST.

Contradictory to our results, Anderson et al. found no difference between the incidence of POST using GL and MCL in morbidly obese patients [13], possibly due to the longer TTI needed by them with GL versus MCL (48 s v.s 32 s, *P* = 0.001). However, the intubations were performed by different categories of health care providers including nurses, residents and anesthesiologists with the experience of merely 20 or more intubations with GL. In our research, we overcome those limitations by having fairly more experienced anesthesiologists compared to them (having >100 intubations with each device) who performed the ET intubations. Using GL, our anesthesiologists successfully accomplished the ET intubations in only 36 s compared to 48 s required by Anderson et al., and therefore, might have caused less trauma to the airway. The results of Anderson et al. study [13] to some extent, are comparable to our results as a longer TTI contributes to a higher incidence of POST due to greater degree of airway mucosal trauma irrespective of the type of device used during ET placement.

Our results show that while using GL, laryngoscopic grades based on C&L classification are lower and view of the glottis based on POGO score is superior in comparison to MCL which results in reduction in TTI, need of external neck manipulations, number of attempts during ET placement leading to reduction in the incidence and severity of POST. Similar to our results, other researchers also found superior view of the glottis [24], lower grades of C&L’s laryngoscopic view and ET intubation to be easier with GL compared to MCL in patients with normal airway [25] and in morbidly obese patients [13]. Despite getting superior view of glottis using VL, Lewis et al. [24] in a Cochrane database systematic review, found no difference in the incidence of POST and the reason of contradiction to our results is probably heterogeneity of the TTI among their studies. Our research would add to the results of the Cochrane database that longer TTI may be a major contributory factor in the causation of POST.

Our data shows that the overall incidence of POST was comparatively less than that of other studies [5, 26, 27]. We speculate that the reason of the overall reduction in the incidence of POST in our research is the contribution of injection dexamethasone, lornoxicam, paracetamol and PCA which were used (as a part of study protocol) for the patients in both the groups [22, 28, 29].

As the use of GL is increasing in patients with normal airway due to improved visualization of glottis and ease of intubation [7, 30], a reduction in the incidence and severity of POST may also be a contributing factor leading to its popularity in routine case. Future large scale studies, using our results as a baseline, may be required to establish contribution of GL in reduction of incidence and severity of POST during its routine use in patients with normal airway.

### Limitations of the study

In this study we used GL and our results may not be applicable to other types of VLs as there are gross differences in the shapes and clinical performance of different kinds of VLs [31]. Secondly, though all the intubations were performed by anesthesia trainee residents with experience of 100 intubations with each device, their skill cannot be compared to skilled anesthesiologists as ET placement using MCL has a significant learning curve [32]. Additionally, for a valid comparison, relatively more experienced anesthesiologists should have been included, especially with MCL. Thirdly, it was not possible to blind the observer due to design of our study and there is a possibility of bias towards a new device. Fourthly, we used C&L classification to grade the view of the glottis using both the devices while it was originally conceived to grade laryngoscopic view based on direct laryngoscopy only [33]; nevertheless many researchers have used this classification for VLs also [34]. Lastly, in our opinion, we should have checked for the presence of blood on the ET tube as the trauma caused by it may not appear on the intubating instrument.

## Conclusion

GL for ET tube placement results in reduction in the incidence and severity of POST compared to MCL during its routine use in patients with normal airway due to probably superior glottic visualization and reduced TTI.

## Abbreviations

C&L: Cormack and Lehane
ET: Endotracheal intubation
GA: General anesthesia
GL: Glidescope
I.D: Internal diameter
i.v.: Intravenous
IDS: Intubation difficulty score
mcg: Microgram
MCL: Macintosh
NIBP: Non-invasive measurement of blood pressure
PACU: Post anesthesia care unit
PCA: Patient controlled analgesia
POGO: Percentage of glottic opening
POST: Postoperative sore throat
s: Second
SPO_2_: Saturation by pulse oximetry
TOF: Train of four
TTI: Time to intubate
VL: Video laryngoscope
