# Correction to: Incidence and severity of postoperative sore throat: a randomized comparison of Glidescope with Macintosh laryngoscope

**DOI:** 10.1186/s12871-018-0575-8

**Published:** 2018-08-17

**Authors:** Mansoor Aqil, Mueen Ullah Khan, Saara Mansoor, Saad Mansoor, Rashid Saeed Khokhar, Abdul Sattar Narejo

**Affiliations:** 1 Department of Anesthesiology, King Saud University Medical City, P.O Box 7805, Riyadh, 11472 Saudi Arabia; 2 College of Medicine, Alfaisal University, Riyadh, Saudi Arabia; 3 Department of Anesthesiology, King Khalid Hospital, Hail, Saudi Arabia

## Correction

We are thankful to Dr. Deepak Gupta (Clinical Assistant Professor Anesthesiology, Wayne State University/Detroit Medical Center) for bringing to our attention a typographical error in our manuscript [1].

Table 2, ROW 12 was wrongly written as:

Number of patients with sore throat (Yes/No)

At 0 h 22/48 (Glidescope), 41/48 (Macintosh), 0.001 (*P* value).

It should be read as: Number of patients with sore throat (Yes/No)

At 0 h 22/48 (Glidescope), 41/29 (Macintosh), 0.001 (*P* value).
